# Streptococcus mitis Cellulitis Progressing to Severe Sepsis, Septic Shock, and Suspected Toxic Shock Syndrome in a Previously Healthy Child

**DOI:** 10.7759/cureus.101677

**Published:** 2026-01-16

**Authors:** Odaly Guignan, Alaa M Khair, Mohamed Z Elsaied, Shaimaa A Youssef, Srikanth R Thathireddy, Mohamed E Abouzeid

**Affiliations:** 1 Pediatric Critical Care, Saudi German Hospital, Dubai, ARE

**Keywords:** cellulitis, septic shock, streptococcus mitis, toxic shock syndrome, viridans streptococci

## Abstract

*Streptococcus mitis* (*S. mitis*), a commensal member of the viridans group streptococci (VGS), is usually low virulence but can rarely cause severe invasive infections such as streptococcal toxic shock syndrome (STSS). We report the case of a previously healthy four-year-old girl who initially presented with atraumatic right-leg pain and rapidly developed extensive cellulitis, severe sepsis, and septic shock. Despite early ceftriaxone therapy, she deteriorated with fever, hypotension, metabolic acidosis, and progressive spreading of erythema. She required paediatric intensive care unit (PICU) admission for aggressive fluid resuscitation and vasoactive support with adrenaline, dobutamine, and later milrinone. Broad-spectrum antibiotics and clindamycin were given for toxin suppression. Echocardiography revealed a reduced ejection fraction (EF) with biventricular dilation, prompting the administration of intravenous immunoglobulin (IVIG) for suspected myocarditis. Blood cultures confirmed *S. mitis* sensitive to multiple antimicrobials. After eight days of intensive care, the patient improved with the resolution of cellulitis and hemodynamic stabilization. This case highlights the rare ability of *S. mitis* to cause toxin-mediated shock in healthy children and emphasizes the importance of early recognition and aggressive management.

## Introduction

Cellulitis is an acute bacterial infection of the deep dermis and subcutaneous tissues, typically caused by β-hemolytic streptococci, most commonly *Streptococcus pyogenes* and less frequently *Staphylococcus aureus*. Cellulitis presents clinically as a warm, erythematous, oedematous, and tender area, usually without purulence or abscess formation. The risk factors for the development of cellulitis include skin breaks, local trauma, diabetes mellitus, venous stasis, lymphedema, and immunosuppression. Early recognition and appropriate systemic antimicrobial therapy are critical to prevent progression to invasive illness such as sepsis and toxic shock syndrome (TSS) [1].

The viridans group streptococci (VGS) include several species, among which *Streptococcus mitis* (*S. mitis*) is a common commensal of the oral cavity, gastrointestinal tract, upper respiratory tract, skin, and female genital tract. Although these organisms are usually of low virulence, they may behave as opportunistic pathogens in immunocompromised hosts, causing bacteremia, endocarditis, or toxic shock-like syndrome. Severe *S. mitis* infections have been described mainly in patients with malignancy or neutropenia or after bone marrow transplantation and are rare in healthy individuals [2].

Streptococcal toxic shock syndrome (STSS) is characterized by the rapid onset of shock and multiorgan dysfunction due to toxin-mediated immune dysregulation. Although classically associated with group A *Streptococcus*, rare cases caused by VGS, such as *S. mitis*, have been documented, including outbreaks in previously healthy adults [3].

## Case presentation

A previously healthy four-year-old girl presented to the outpatient clinic with isolated atraumatic right-leg pain and inability to fully extend the knee for one day, without fever, erythema, swelling, or systemic symptoms, following playground activity. Orthopaedic assessment excluded fracture, and she was diagnosed with hamstring strain and treated conservatively with topical gel and crepe bandage. The next day, she developed an acute onset of high-grade fever, 38.9°C, worsening leg pain, redness, and swelling along the internal aspect of the right calf. She was brought to the emergency department 24 hours later complaining of persistent fever, progression of the erythema, and three episodes of vomiting. She was admitted to the paediatric ward with the diagnosis of cellulitis of the right lower limb, fever, vomiting, and dehydration. Ceftriaxone was initiated, but after 48 hours of evolution, her condition deteriorated, with rising C- reactive protein (CRP) (203 mg/L) (Table 1).

**Table 1:** Summary of serial laboratory results during hospitalization RBC: red blood cell count; MCV: mean corpuscular volume; MCH: mean corpuscular hemoglobin; MCHC: mean corpuscular hemoglobin concentration; RDW: red cell distribution width; WBC: white blood cell count; ESR: erythrocyte sedimentation rate; CK: creatine kinase; CK-MB: creatine kinase myocardial band; TnThs: troponin T-high sensitivity; NT-proBNP: N-terminal pro-B-type natriuretic peptide; IgA: immunoglobulin A; IgM: immunoglobulin M; IgG: immunoglobulin G

Serial laboratory/date	03-Nov-2011	04-Nov-2011	05-Nov-2011	06-07-Nov-2011	08-Nov-2011	09-Nov-2011
RBC count	5.18x10^12^/L	3.92x10^12^/L	4.33x10^12^/L	3.80x10^12^/L	3.93x10^12^/L	4.36x10^12^/L
Hemoglobin	13.7 g/dL	10.6 g/dL	11.2 g/dL	10.1 g/dL	10.5 g/dL	11.4 g/dL
Hematocrit	40.90%	30.80%	34.40%	31.40%	30.40%	34.20%
MCV	78.9 fL	78.5 fL	79.4 fL	82.4 fL	77.4 fL	78.4 fL
MCHC	33.5 g/dL	34.5 g/dL	32.6 g/dL	32.4 g/dL	34.6 g/dL	33.3 g/dL
MCH	26.4 pg	27.1 pg	25.9 pg	26.7 pg	26.8 pg	26.1 pg
RDW	13.10%	13.10%	14.40%	14.40%	14.10%	14.50%
Platelet count	279x10^9^/L	157x10^9^/L	48x10^9^/L	48x10^9^/L	77x10^9^/L	183x10^9^/L
Mean platelet volume	8.1 fL	8.1 fL	8.0 fL	8.0 fL	9.0 fL	9.6 fL
WBC count	11.10x10^9^/L	14.00x10^9^/L	13.20x10^9^/L	13.20x10^9^/L	9.60x10^9^/L	12.7x10^9^/L
Neutrophils %	86.40%	95.00%	979.20%	979.20%	31.00%	50.50%
Lymphocytes %	10.50%	1.20%	12.10%	12.10%	57.00%	37.90%
Monocytes %	2.70%	1.60%	6.70%	6.70%	6.30%	9.40%
Eosinophils %	0.20%	1.80%	1.90%	1.90%	5.20%	1.80%
Basophils %	0.20%	0.40%	0.10%	0.10%	0.50%	0.40%
Absolute neutrophil count	9.6x10^9^/L	13.3x10^9^/L	10.4x10^9^/L	10.4x10^9^/L	3.0x10^9^/L	6.4x10^9^/L
Absolute lymphocyte count	1.2x10^9^/L	0.2x10^9^/L	1.6x10^9^/L	1.6x10^9^/L	5.5x10^9^/L	4.8x10^9^/L
Absolute monocyte count	0.3x10^9^/L	0.2x10^9^/L	0.9x10^9^/L	0.9x10^9^/L	0.6x10^9^/L	1.2x10^9^/L
Absolute eosinophil count	0.0x10^9^/L	0.2x10^9^/L	0.3x10^9^/L	0.3x10^9^/L	0.5x10^9^/L	0.2x10^9^/L
C-reactive protein	6 mg/L	203 mg/L	187 mg/L	93 mg/L	-	18 mg/L
Prothrombin time	18.7 sec	-	28 sec	19.8 sec	-	-
International normalized ratio	1.4	-	2.2	1.5	-	-
Activated partial thromboplastin	30.4 sec	-	33.9 sec	35.1 sec	-	-
ESR	-	20 mm/H	-	-	-	-
CK	-	-	-	-	13 U/L	-
CK-MB	-	-	-	-	1.53 ng/mL	-
TnThs	-	-	-	-	64 ng/L	42 ng/L
NT-proBNP	-	-	-	-	>35000 pg/mL	2646 pg/mL
D-dimer	-	-	-	> 20 FEU ug/mL	-	-
IgA total antibody	-	-	-	-	62 mg/dL	-
IgM total antibody	-	-	-	-	75 mg/dL	-
IgG total antibody	-	-	-	-	499 mg/dL	-

The patient developed increasing pain, with progressive extension of erythema to the knee and ankle. She developed tachycardia, poor perfusion, a capillary refill time (CRT) of five seconds, and emerging signs of shock. She was transferred to the paediatric intensive care unit (PICU) with severe sepsis and septic shock, prompting fluid resuscitation and the initiation of adrenaline infusion (0.05 µg/kg/min) by peripheral vascular line. Additional empiric antibiotics, namely, vancomycin and meropenem, were started. The venous blood gas demonstrated a pH of 7.28 and an HCO₃⁻ of 17 mmol/L, consistent with metabolic acidosis. Doppler ultrasound excluded compartment syndrome, with preserved arterial and venous flow. Consultation with an orthopaedic doctor was done, and a magnetic resonance imaging (MRI) of the limb was recommended.

Over the following hours, the erythema rapidly extended up the thigh, and due to suspicion of STSS, clindamycin was added for toxin suppression. Initial echocardiogram showed an ejection fraction (EF) of 78%, no effusion, and normal valves.

Despite therapy, she developed worsening hypotension, respiratory distress, and dilated inferior vena cava (IVC) with low collapsibility, requiring the escalation of inotropes to adrenaline 0.2 µg/kg/min and dobutamine 10 µg/kg/min via a newly placed left internal jugular central venous catheter. She required high-flow oxygen (30 L/min) and a fraction of inspired oxygen (FiO₂) of 100%.

Repeat echocardiography showed a decrease in the left ventricular (LV) function, compared to the previous echo, an EF of 50%, a fractional shortening (FS) of 38%, IVC dilation, mild mitral regurgitation, and normal aortic and pulmonary flow. The abdomen became distended with hepatomegaly 5 cm below the costal margin. Laboratory findings included leucocytosis, neutrophilia, elevated D-dimers, hypoalbuminemia, and persistent normal anion gap metabolic acidosis with hyperchloremia.

**Figure 1:**
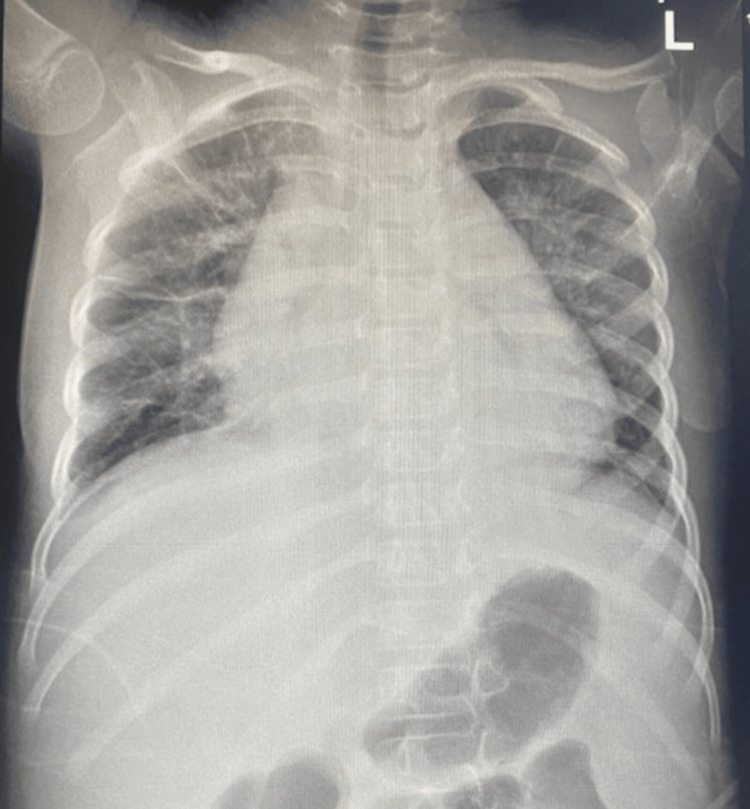
Chest X-ray day 4 of PICU admission PICU: paediatric intensive care unit

On PICU day 4, she developed worsening respiratory distress. Chest X-ray showed an enlarged cardiac silhouette (Figure 1).

**Figure 2:**
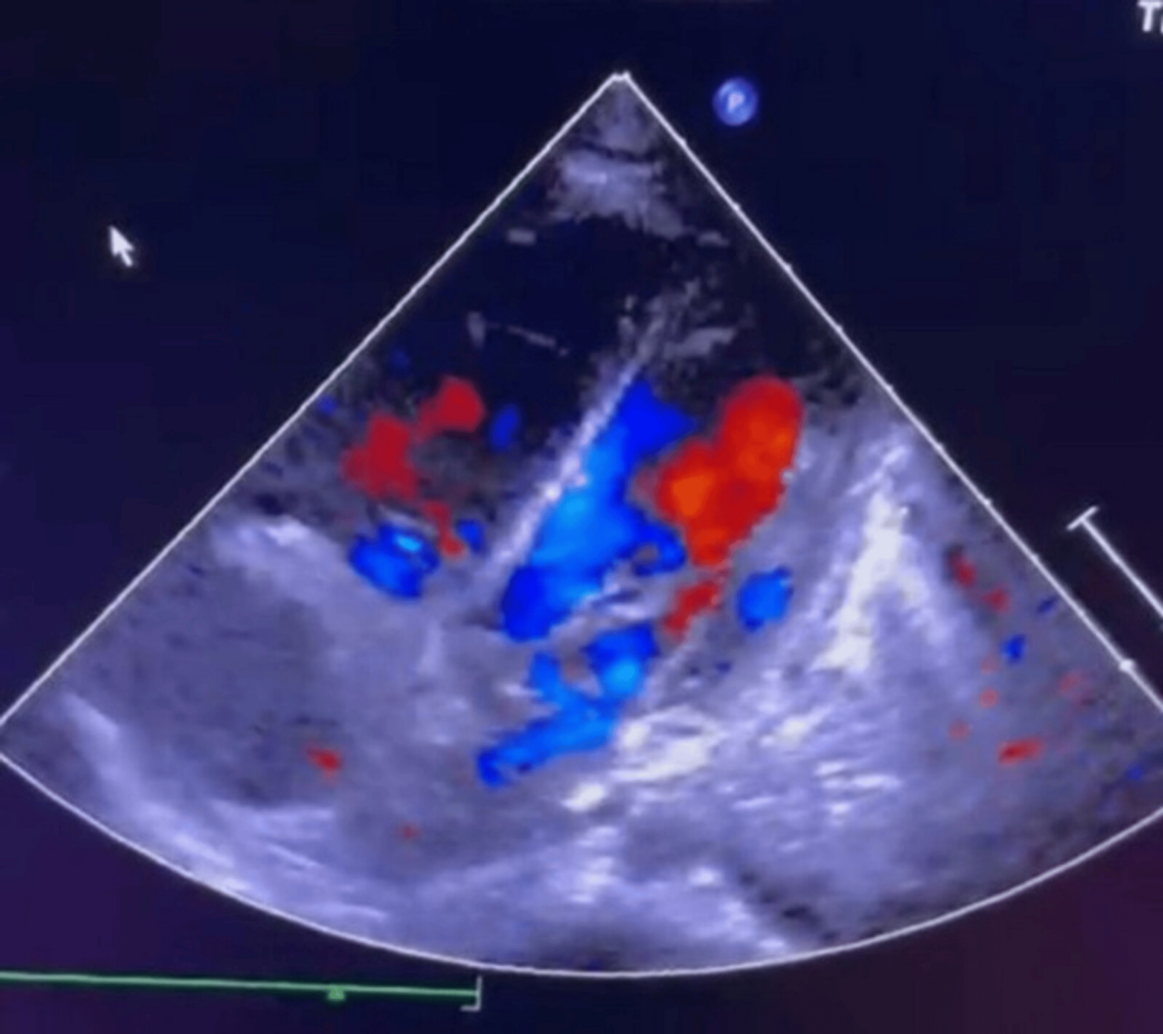
Echocardiogram showing biventricular dilatation mainly in the right side

The echocardiogram revealed biventricular dilation, predominantly on the right, moderate tricuspid regurgitation (36-40 mmHg), mild mitral regurgitation, and an EF of 45%, raising suspicion of myocarditis related to septic shock or STSS (Figure 2).

Troponin and N-terminal pro-B-type natriuretic peptide (NT-pro-BNP) were markedly elevated. She received intravenous immunoglobulin (IVIG) (2 g/kg). Computed tomography pulmonary angiogram (CTPA) was done to exclude pulmonary embolism, and it showed pleural effusion, ascites, cardiomegaly, and enlarged axillary lymph nodes.

Inotropic support was adjusted to milrinone 0.25 µg/kg/min, with furosemide 1 mg/kg three times a day, and fluid restriction (80%).

The final blood culture identified *S. mitis*, which was sensitive to ceftriaxone, vancomycin, clindamycin, and penicillin. After eight days in the PICU, the child improved significantly, with the regression of the cellulitis and the stabilization of cardiovascular and respiratory status. A multidisciplinary team involving pediatricians, an intensivist, orthopedic and pediatric surgery teams, a dietician, rehabilitation specialists, and a physiotherapist was involved in the patient's management.

## Discussion

This case illustrates an uncommon progression of *S. mitis* cellulitis to severe sepsis, septic shock, toxic shock-like syndrome, and suspected myocarditis in a previously healthy child. While cellulitis is typically caused by β-hemolytic streptococci or *Staphylococcus aureus*, VGS infections are unusual in immunocompetent hosts. Breaks in the skin barrier may facilitate bacterial invasion into the dermis and subcutaneous tissue, triggering a local inflammatory response and potentially progressing to bacteremia and systemic toxicity [1].

*S. mitis *is a commensal organism but can produce superantigen-like toxins, inducing massive cytokine activation, vascular leak, and multiorgan dysfunction characteristic of STSS. Such presentations remain rare but have been described in outbreaks and in isolated case reports in adults and immunocompromised individuals [2-4].

VSG species colonize the oropharynx, gastrointestinal tract, and genitourinary tract, and their microbiological diversity and variable hemolytic patterns make classification challenging. *S. mitis *is typically associated with immunocompromised hosts, particularly those with malignancy or neutropenia, and is among the VGS species most frequently isolated in the majority of the blood cultures of oncologic patients. Guerrero-Del-Cueto et al. reported in a study in oncology patients that *S. mitis *was responsible for severe primary bacteremia in up to 80% of cancer patients in the Shelburne cohort, with high disease severity scores. However, unlike those populations, our patient had no underlying immunodeficiency, highlighting the unusual and unexpected virulence displayed by *S. mitis *in this case. This underscores the organism's potential to cause severe toxin-mediated shock even in previously healthy children [5].

The clinical course of our patient is consistent with the known challenges of recognizing paediatric sepsis in its early stages. As described by Miranda and Nadel, paediatric sepsis remains a major global health problem and accounts for more than 8% of PICU admissions, with over 4.5 million childhood deaths annually. Early recognition of sepsis and its progression is often difficult because febrile illnesses are common in children, clinical features are nonspecific, and paediatric patients can physiologically compensate until shock is already advanced. This pattern was evident in our case, as the child initially presented with isolated leg pain and localized cellulitis but rapidly progressed to severe sepsis and septic shock despite early antibiotic therapy. The subtle early signs, followed by sudden decompensation with hypotension, poor perfusion, and metabolic acidosis, illustrate the diagnostic challenges highlighted in the literature and emphasize the need for heightened clinical vigilance even in children without underlying comorbidities [6].

The patient's evolution from localized cellulitis to shock within 48 hours highlights the aggressiveness of toxin-mediated streptococcal disease. STSS diagnostic criteria include hypotension and multiorgan involvement and renal, hepatic, coagulopathic, respiratory, or soft tissue necrosis [7].

Management follows the Surviving Sepsis Guidelines, emphasizing early, broad-spectrum antibiotics within one hour, source control, fluid resuscitation, vasoactive support, and toxin-suppressing antibiotics such as clindamycin. Clindamycin reduces bacterial toxin production and improves outcomes in streptococcal invasive infections [8].

During deterioration, the child developed worsening signs of shock with tachycardia, poor perfusion, and hypotension, requiring the prompt initiation of epinephrine infusion at 0.05 µg/kg/min, consistent with paediatric septic shock management. In accordance with current recommendations, vasoactive medications were started through a peripheral intravenous line, as treatment should not be delayed while obtaining central access in critically ill children. Epinephrine is preferred in paediatric septic shock when myocardial dysfunction or low cardiac output states are suspected. Placement of a central venous catheter may be delayed in patients anticipated to stabilize and wean off vasoactive medication within a short timeframe; however, due to ongoing hemodynamic instability, parental consent was obtained, and the central line was ultimately inserted to facilitate escalating support. This approach aligns with evidence demonstrating the safety of peripheral or intraosseous vasoactive infusions in paediatric critical care when carefully monitored [9,10].

IVIG may neutralize streptococcal superantigens in severe STSS, although evidence remains mixed. In this case, its use coincided with clinical improvement. For severe nonpurulent cellulitis, the Infectious Diseases Society of America (IDSA) guidelines recommend penicillin or clindamycin, with broader coverage (vancomycin+piperacillin/tazobactam or a carbapenem) for severe systemic illness [11].

The echocardiographic abnormalities observed in this case are consistent with patterns described in the systematic review by Schmutzler et al., which evaluated myocarditis following streptococcal pharyngitis or tonsillitis. In their review, 10% of patients demonstrated a reduced LVEF of <55% at admission, similar to our patient who experienced a decline from 78% to 45-50% during the peak of illness. Additionally, regional wall motion abnormalities were common in the reviewed cases (21.4%), and our patient also developed biventricular dilation and functional impairment consistent with myocardial involvement. Unlike the review, which reported mild pericardial effusion in 7.1%, our patient had no pericardial effusion throughout evaluation. Valvular involvement was rare in the review (1.4% with moderate mitral regurgitation), and our patient likewise exhibited only mild mitral and moderate tricuspid regurgitation, likely secondary to acute ventricular dysfunction rather than structural disease. These findings further support the diagnosis of myocarditis secondary to severe streptococcal infection [12].

Myocardial involvement in severe sepsis and STSS is well described, mediated by inflammatory injury, cytokine toxicity, and microvascular dysfunction.

Echocardiographic findings in this child, including a reduced EF, chamber dilation, and elevated biomarkers, were consistent with septic cardiomyopathy and toxin-associated myocarditis, both reversible with appropriate management [12,13].

Overall, this case underscores the need for high clinical suspicion when cellulitis rapidly progresses or fails conventional therapy, especially when accompanied by shock, multiorgan involvement, or toxin-mediated features. Early multidisciplinary management is essential to reduce morbidity and mortality.

## Conclusions

This case demonstrates the exceptional severity that *S. mitis* may achieve in an immunocompetent child, progressing rapidly from simple cellulitis to severe sepsis, septic shock, systemic toxin effects, and suspected myocarditis. Although VGS are normally low-pathogenicity commensal organisms, clinicians must remain vigilant when infections fail standard therapy or exhibit unusually rapid extension. Early aggressive management following paediatric sepsis guidelines, including broad-spectrum antibiotics, hemodynamic stabilization with vasoactive agents, and adjunctive toxin-suppressing therapy such as clindamycin, is essential to prevent mortality. The clinical improvement observed after IVIG suggests a potential therapeutic role in toxin-mediated disease. This case reinforces that rare pathogens such as *S. mitis* can produce life-threatening illness even in healthy children and highlights the importance of timely diagnosis, multidisciplinary care, and individualized escalation of treatment.
